# The Outcome- or Cost-Effectiveness Analysis of LUS-Based Care or CXR-Based Care of Neonatal Lung Diseases: The Clinical Practice Evidence from a Level Ⅲ NICU in China

**DOI:** 10.3390/diagnostics12112790

**Published:** 2022-11-14

**Authors:** Jing Liu, Xin Zhang, Yan Wang, Jie Li, Wei Yan, Sheng-Juan Qin, Xiao-Ling Ren, Wei Fu

**Affiliations:** 1 Department of Neonatology and NICU, Beijing Chao-Yang Hospital, Capital Medical University, Beijing 100043, China; 2 Department of Neonatology and NICU, Zhumadian Central Hospital of Henan Province, Zhumadian 463003, China; 3 Department of Neonatology and NICU, The Affiliated Taian City Central Hospital of Qingdao University, Taian 271000, China; 4 Department of Neonatology and NICU, Zaozhuang Maternal and Child Healthcare Hospital of Shandong Province, Zaozhuang 277100, China; 5 Department of Ultrasound, Zhumadian Central Hospital of Henan Province, Zhumadian 463003, China; 6 Department of Neonatology and NICU, Beijing Chao-Yang District Maternal and Child Healthcare Hospital, Beijing 100021, China

**Keywords:** lung ultrasound (LUS), lung disease, newborn infants, chest X-ray (CXR), outcome-effectiveness or cost-effectiveness, health authorities

## Abstract

Objective: To compare the effect of managing neonatal lung disease with lung ultrasound (LUS) or chest X-ray (CXR) monitoring on health outcomes and cost-effectiveness. Methods: The data obtained from the NICU of the Beijing Chaoyang District Maternal and Child Healthcare Hospital were used as the study group, as LUS has completely replaced CXR in managing newborn lung disease in the hospital for the past 5 years. The primary outcomes of this study were the misdiagnosis rate of respiratory distress syndrome (RDS), the using status of mechanical ventilation, the incidence rate of bronchopulmonary dysplasia (BPD) and the survival rate in hospitalized infants. The secondary outcomes included the use pulmonary surfactant (PS), and the mortality rate of severe diseases (such as pneumothorax, pulmonary hemorrhage and RDS, etc.). Results: Managing neonatal lung disease with LUS monitoring may enable the following effects: The frequency of ventilator use reducing by 40.2%; the duration of mechanical ventilation reducing by 67.5%; and the frequency of ventilator weaning failure being totally avoided. A misdiagnosis rate of 30% for RDS was also avoided. The dosage of PS was significantly reduced by 50% to 75%. No BPD occurred in the LUS-based care group for 5 years. The fatality rates of RDS, pneumothorax and pulmonary hemorrhage decreased by 100%. The poor prognosis rate of VLBW infants decreased by 85%, and the total mortality rate of hospitalized infants decreased by 90%. Therefore, the cost of LUS-based care was inevitably saved. Conclusions: Diagnosing and managing neonatal lung diseases with LUS monitoring have significant benefits, and this technology should be widely promoted and applied around the world.

## 1. Introduction

Lung disease is the leading and most common cause of hospitalization and death among newborn infants, and its diagnosis relies on chest X-ray (CXR) examination. However, CXR examination has certain limitations, such as a high misdiagnosis rate, low sensitivity, poor specificity and inevitable radiation damage, especially among developing premature infants and newborns, who are considerably more susceptible to ionizing radiation because the rate at which their cells undergo mitosis is more rapid than that observed in adult populations [1]. Increased radiosensitivity, greater mitotic activity and a protracted period for consequences to manifest lead to a 2- to 3-fold higher risk of radiation-induced cancer per unit of dose among preterm infants than among the average population [2]. A much recent long-term follow-up study showed that for every 10 mGy increase in the dose of radiation received in childhood, the risk of central nervous system tumors increased 1.05 times (95% CI: 1.01–1.09), and the risk of leukemia was increased by 1.17 times (95% CI: 1.09–1.26) [3]. In recent years, using ultrasound to diagnose neonatal lung diseases has been widely used in clinical practice [4,5,6,7,8,9,10,11]. Lung ultrasound (LUS) is more convenient, and can be performed faster than traditional CXR, with higher sensitivity, accuracy, specificity and reliability [11,12], and it protects both newborn infants and medical staff from radiation damage. As a result, LUS has replaced CXR for the past five years in the diagnosis and management of neonatal lung disease (named LUS-based care in this paper) in our neonatal intensive care unit (NICU) [13]. It greatly improves the prognosis of newborn infants, and provides dramatic social and economic benefits.

In the present study, we investigate and compare the possible impact and benefits of LUS-based care or traditional use of CXR to diagnose and manage lung disease in newborn infants (CXR-based care) in the NICU of public health institutions; thus, an outcome-effectiveness or cost-effectiveness analysis was conducted in this paper. Therefore, the analysis can serve as a reference for health management departments in the health management decision-making process, as well as provide strong evidence for the routine implementation of LUS in the NICU.

## 2. Objects and Methods

### 2.1. Ethics Approval

This work was approved by the Ethics Committee of the Ethics Committee of Beijing Chaoyang District Maternal and Child Healthcare Hospital (No. 2011-LC-Ped-01).

### 2.2. Objects

**Figure 1:**
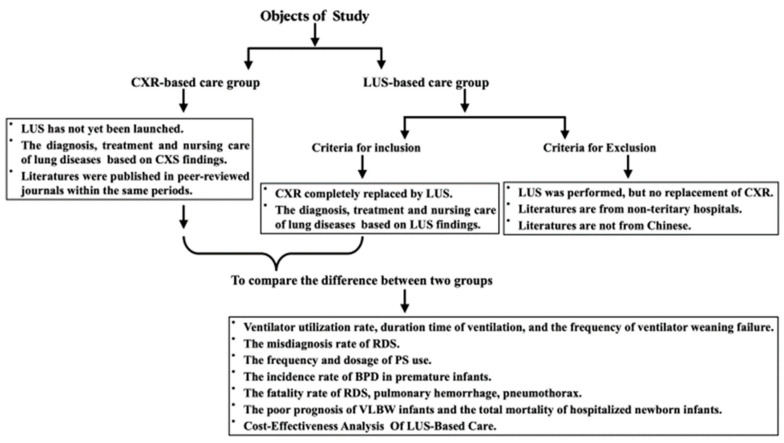
The flow chart of the data and cases selection methods.

The data obtained from the NICU of Beijing Chaoyang District Maternal and Child Healthcare Hospital were used as the study group (LUS-based care group) because LUS has completely replaced CXR in managing (including lung diseases diagnosed by LUS, lung diseases treatment guided under LUS and precision nursing care of lung diseases under LUS monitoring) newborn lung disease in the hospital for the past 5 years [13], while the data from hospitals that have not performed LUS and still use CXR to diagnose neonatal lung disease or data published in peer-reviewed Chinese journals in the last 5 years were used as controls (CXR-based care group, that is that the diagnosis, treatment and nursing care of lung diseases are based on CXR findings). The criteria for the controls (CXR-based care group) must meet the following criteria: (1) Neonatal LUS has not been performed, and the diagnosis and management of neonatal lung diseases still rely on the use of CXR. (2) Literature was published in peer-reviewed journals within the last 5 years. (3) For the dosage of exogenous pulmonary surfactant (PS), the dosage of the internationally recognized European respiratory distress syndrome (RDS) guideline recommended was used as the control [14] (Figure 1). Exclusion criteria: (1) Although LUS has been introduced in some hospitals, it has not replaced X-rays. Therefore, information from these hospitals was not included in this study. (2) Literature is from non-tertiary hospitals. (3) To ensure that the data of the two groups is comparable and to eliminate errors caused by unequal economic and technological development as much as possible, the data of the control group were all from China. Therefore, relevant literature from other countries was not included in this study (Figure 1).

### 2.3. Observation Index

(1) Influence on ventilator utilization rate, duration time of ventilation and the frequency of ventilator weaning failure. (2) Influence on the misdiagnosis rate of RDS. (3) Influence on the frequency and dosage of PS use. (4) Influence on the incidence rate of bronchopulmonary dysplasia (BPD) in premature infants. (5) Effect on the fatality rate of RDS. (6) Effect on the fatality rate of pulmonary hemorrhage. (7) Effect on the pneumothorax fatality rate. (8) Effects on the poor prognosis of very-low-birth weight (VLBW) infants. (9) Effect on the total mortality of hospitalized newborn infants (Figure 1).

### 2.4. Statistical Methods

SPSS 24.0 software (IBM Inc.; Armonk, NY, USA) was used for statistical processing. The chi-squared test or Fisher’s exact test was used to compare the rates of each indicator between the two groups, and Student’s *t* test was used to compare the mean values of the two samples. *p* < 0.05 was considered statistically significant.

## 3. Results

### 3.1. Influence on Ventilator Utilization Rate, Duration Time of Ventilator and the Frequency of Ventilator Weaning Failure

(1) Among the infants admitted to our NICU during the last 5 years, 597 cases (11.9%) needed invasive ventilator treatment, according to CXR-based care, among a total of 5027 hospitalized newborns, while only 357 cases (7.1%) received invasive ventilator treatment by LUS-based care [15], in a decrease of 40.2% compared with CXR-based indications. (2) The total average duration time of mechanical ventilation was 4.88 days among 357 patients who received LUS-based care, while it was 15.03 days among CXR-based care in 672 patients. LUS-based care reduced the duration of invasive ventilator treatment by 67.5% [Table 1]. (3) Two domestically qualified studies about the repeated use of ventilators in neonates were retrieved, in which the frequency of repeated use of ventilators was 16.7% (68/408) [16,17]. Under the guidance of LUS monitoring, among the 357 infants who received ventilator treatment, none of them went on the ventilator again due to ventilator weaning failure; therefore, the repetition rate was 0%, which decreased by 100% compared with the traditional method.

### 3.2. Influence of LUS-Based Care on the Misdiagnosis Rate of RDS

According to the literature, the traditional use of CXR to diagnose RDS has resulted in a misdiagnosis rate of more than 62% [18]. In particular, transient tachypnea of newborns (TTN) has been frequently misdiagnosed as RDS [18,19]. In the last 5 years, 385 newborn infants admitted to our NICU met the traditional diagnostic criteria of RDS, but only 269 cases were ultimately diagnosed with RDS after LUS examination [20,21,22,23,24]. An additional 116 cases were diagnosed with TTN in 82 cases [22,23,24,25], pneumonia in 21 cases [22,26] and meconium aspiration syndrome (MAS) in 13 cases [22,27]. That is, LUS-based care prevented 30% of misdiagnosed cases of RDS. We can see from the following two cases that their LUS findings were very different from each other, even though they had many similar clinical characteristics.

**Figure 2:**
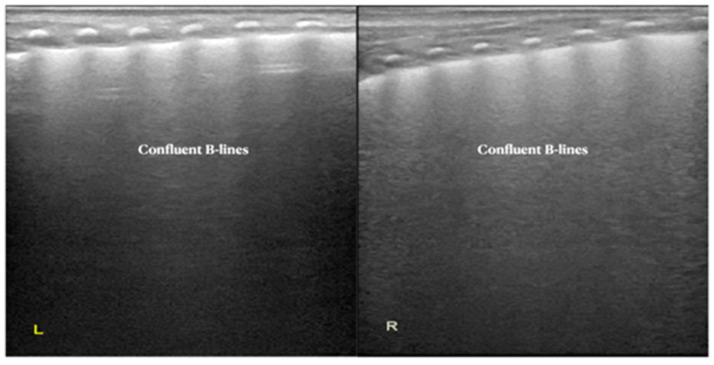
LUS findings of Case 1 infant. LUS presented as confluent B-lines confirmed as TTN (L/R: left/right lung).

Case 1: This male baby was G_1_P_1_ with a gestational age of 34^+5^ weeks, cesarean delivery and birth weight of 2370 g. The infant was admitted to NICU because of progressive dyspnea and expiratory moans after 20 min of birth. Significant retraction was noted at physical examination [Video S1]. Arterial blood gas analysis showed PaCO_2_ 65.3 mmHg, PaO_2_ 52 mmHg and SaO_2_ 77%. According to the case history, clinical manifestation and arterial blood gas results were clinically consistent with RDS features, but the LUS showed significantly confluent B-lines in bilateral lungs, which is the typical LUS characteristic of TTN rather than RDS [Figure 2]. 

**Figure 3:**
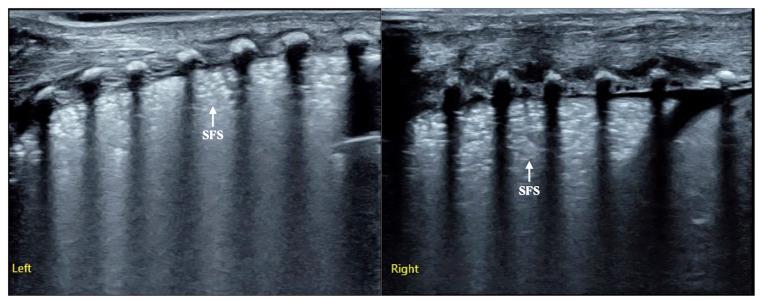
LUS findings of Case 2 infant. LUS presented as snowflake-like sign consolidation confirmed as RDS (Left: left lung, Right: right lung).

Case 2: This female baby was G_1_P_1_ with a gestational age of 31^+4^ weeks, vaginal delivery, and a birth weight of 1880 g. She was admitted to our NICU because of progressive dyspnea, significant expiratory moans and retraction for 1 h [Video S2]. Arterial blood gas analysis showed PaCO_2_: 56 mmHg, PaO_2_: 42 mmHg and SaO_2_: 79%. On admission, LUS examination showed snowflake-like sign (SFS) lung consolidation in the bilateral lungs, which was consistent with the typical ultrasound imaging characteristics of RDS [Figure 3].

### 3.3. Influence of LUS-Based Care on the Frequency and Dosage of PS

As mentioned above, among the 385 cases diagnosed with RDS according to the traditional criteria, only 269 cases were diagnosed with RDS, according to the LUS diagnostic criteria [20,21,22]. The 114 cases without LUS diagnosis were not treated with PS; therefore, the application of PS was reduced by 30%.

In the LUS-based care groups, the first dosage of PS was 100 mg/kg, and the repeated dosage was only 50 mg/kg per time, while the dosage was 200 mg/kg recommended by the European RDS management guidelines (China follows these guidelines) [14]. As a result, the initial dosage was reduced by 50%, and the repeated dosage was reduced by 75% under LUS-based care compared to the dosage recommended by the European RDS management guidelines.

### 3.4. Influence of LUS-Based Care on the Incidence of BPD in Premature Infants

In the present study, the diagnostic criteria for BPD followed the criteria released by the National Institute of Child Health and Human Development (NICHD) in 2001 [28]. A total of 11 studies were retrieved that met the inclusion criteria of this study [29,30,31,32,33,34,35,36,37,38,39]. Among them, there were 5 studies on very extremely premature infants (with gestational age < 28 weeks), including 959 cases of BPD among the 1212 cases of very extremely premature infants; therefore, the incidence rate of BPD was as high as 79.1% [29,30,31,32,33]. There were 5 reports on extremely premature infants (with gestational age < 32 weeks), including 357 cases of BPD among a total of 917 cases of extremely premature infants, and the incidence rate of BPD was 38.9% [34,35,36,37,38]. There was 1 study on middle- to late-preterm infants, in which the incidence rate of BPD in middle-preterm infants (gestational age of 32 to 34 weeks) was 1.63%, and that of late-preterm infants (gestational age 34 to 37 weeks) was 0.31% [39]. In the past 5 years, a total of 1033 premature infants were admitted to our NICU, none of whom developed BPD; thus, the incidence rate of BPD was 0%. This means that LUS-based care of premature infants reduced the incidence rate of BPD by 100%.

### 3.5. Effect of LUS-Based Care on the Fatality Rate of RDS

A total of 3 studies that met the inclusion criteria of this paper that studied the fatality rate of RDS were retrieved [40,41,42]. A total of 282 cases of RDS were included in the literatures, including 40 deaths, resulting in a fatality rate of 14.2%. As mentioned above, a total of 269 cases of RDS were diagnosed in our NICU in the last 5 years, all of whom were cured and safely discharged, and the fatality rate of RDS was 0% (*x*^2^ = 42.32, *p* < 0.001) [Table 2]. That is to say, all RDS can be cured, and the fatality rate decreased by 100% under LUS-based care.

### 3.6. Effect of LUS-Based Care on the Fatality Rate of Pulmonary Hemorrhage

A total of 2 studies that met the inclusion criteria of this paper that studied the fatality rate of pulmonary hemorrhage were retrieved [43,44]. A total of 245 cases of pulmonary hemorrhage were reported in the two studies, including 79 deaths, resulting in a fatality rate of 32.2%. A total of 82 cases of pulmonary hemorrhage were diagnosed in our NICU in the last 5 years, all of which were cured and safely discharged, and the fatality rate of pulmonary hemorrhage was 0% (*x*^2^ = 34.86, *p* < 0.001) [Table 3]. That is to say, the fatality rate decreased by 100% under LUS-based care.

### 3.7. Effect of LUS-Based Care on the Fatality Rate of Pneumothorax

A total of 4 studies that met the inclusion criteria of this paper and studied the fatality rate of pneumothorax were retrieved [45,46,47,48]. A total of 373 cases of pneumothorax were reported in the three studies, including 41 deaths, resulting in a fatality rate of 11.0%. A total of 61 cases of pneumothorax were diagnosed in our NICU in the last 5 years, all of which were cured and safely discharged, and the fatality rate of pneumothorax was 0% (*x*^2^ = 7.41, *p* = 0.007) [Table 4]. That is to say, the fatality rate decreased by 100% under LUS-based care.

### 3.8. Effect of LUS-Based Care on the Prognosis of Very Low Birth Weight (VLBW) Infants

According to data from the Collaborative Quality Improvement Team of Neonatal Intensive Care Units in China, among 2956 VLBW infants with birth weights of <1500 g, 1373 infants had a poor prognosis (including death, and their parents voluntarily stop the treatment, mostly due to severe illness), with a poor prognosis rate of 46.5% in 25 tertiary NICUs in China [49]. A total of 81 VLBW infants were diagnosed in our NICU in the last 5 years; 5 of these had a poor prognosis, and the overall poor prognosis rate was 6.2% (*x*^2^ = 51.60, *p <* 0.001) [Table 5]. The poor prognosis rate of VLBW infants decreased by 85.2% under LUS-based care.

### 3.9. Effect of LUS-Based Care on the Total Mortality of Hospitalized Patients

A total of 5 studies that met the inclusion criteria of this study concerning the mortality rate of hospitalized patients were retrieved [50,51,52,53,54]. A total of 126,565 hospitalized patients were included in the literature, including 1244 deceased infants, resulting in a mortality rate of 9.83‰. A total of 5027 hospitalized patients in our NICU in the last 5 years, 5 of whom died, resulted in a total mortality of 0.99‰ (*x^2^* = 40.14, *p* < 0.001) [Table 6]. The mortality rate of hospitalized patients decreased by 90% under LUS-based care.

## 4. Discussion

The results of this study indicate that using LUS instead of CXR in the diagnosing and guiding the management of neonatal lung disease has many advantages, including increased diagnostic efficiency, reduced misdiagnosis and improved therapeutic effects. LUS can also preserve medical resources, shorten the hospital stay and reduce the hospitalization cost of newborn infants. In particular, it can prevent the occurrence of premature BPD, greatly improve the treatment success rates of RDS, pulmonary hemorrhage, pneumothorax and other serious lung diseases, significantly reduce the mortality rate of hospitalized infants, and achieve significant social and economic benefits. The discussion is based on the following aspects.

### 4.1. Reducing the Frequency of Ventilator Use, Shortening the Duration of Ventilation, and Avioding the Ventilator Weaning Failure

Mechanical ventilation is one of the most essential and important measures for the treatment of newborn infants with dyspnea. The development of LUS has confirmed that traditional machine indications are not reliable, and ventilators under LUS guidance can not only significantly reduce the ventilator frequency, but also significantly shorten the ventilator duration [15]. The results of this study showed that guiding ventilator application under LUS monitoring has the following advantages: (1) Greatly reducing the invasive ventilator rate: LUS-based care decreased by 44.4% compared with CXR-based care. (2) Significantly shortening the ventilator duration time: for those patients who still needed to use the ventilator, the ventilator duration time was shortened by 64.7% compared with the traditional one. (3) LUS-based care effectively avoiding repeated ventilator use: according to the literature, the repeated ventilator use rate in China is 15.3–20% [16,17], and the failure rate of ventilator withdrawal in extremely premature infants is as high as 32–50% [55]. We weaned ventilator use under LUS monitoring; thus far, there has been no repeat use of the ventilator. (4) There are also many complications associated with ventilator use, such as ventilator-associated pneumonia, pneumothorax, emphysema, sepsis, and BPD [56,57]. In addition, as the duration of ventilation and the number of repetitions increase, the incidence rates of the complications mentioned above also increase [55,56,57]. However, ventilator withdrawal under LUS monitoring can reduce the occurrence of these complications.

### 4.2. Avoiding RDS Misdiagnosis and Reducing PS Dosage

PS is one of the most important agents for the treatment of neonatal dyspnea, especially for RDS, is widely used in clinical practice due to its significant effect and is one of the key recommended treatment measures in the RDS management guidelines [14,58,59]. The advantages of LUS-based care in this aspect include the following: (1) Significantly reducing the misdiagnosis of RDS: According to the traditional understanding of the disease and traditional diagnostic criteria, TTN is easily misdiagnosed as RDS, especially when an infant with dyspnea is premature, dyspnea is progressively aggravated and accompanied by expiratory groans, there are serious abnormalities in arterial blood gas analysis, and CXR shows “white lung” or close to “white lung”. These manifestations are considered the “gold standard” for the diagnosis of RDS [18,19]. Therefore, it is easy for clinicians to diagnose these newborn infants as RDS and administer PS treatment, which leads to the expansion of the application of PS. However, LUS can easily distinguish TTN from RDS [10,11,12,13,14,15,16,17], avoiding 30% misdiagnosis and significantly reducing the probability of PS use. According to statistics, the incidence of RDS accounts for 1.72–8.2% of all live births, including 23.8–37.3% of premature infants and 1.64% of full-term infants [60,61]. The incidence of premature infants is on the rise worldwide, and by 2016, the incidence of premature infants in China had exceeded 10% [62], and was even as high as 17.1% [63]. Based on the annual birth rate of 12 million newborns in China, according to traditional diagnostic standards, there are at least 500,000 to 750,000 RDS babies in China every year. Under LUS diagnosis, at least 150,000~225,000 misdiagnoses can be reduced. (2) Significantly reducing the dosage of PS: at present, in many countries, including China, the dosage of PS used for premature RDS is 200 mg/kg per dose, as recommended by the European RDS management guidelines [14]. Our experience shows that this dosage is significantly higher, and combined with its high cost, undoubtedly increases the cost of treatment for newborn infants. We used PS under LUS monitoring, and the first dosage was 100 mg/kg. If repeated application is required, the dosage of repeated use is 50 mg/kg. This dosage also worked well. Therefore, the use of PSs under LUS monitoring can greatly reduce the hospitalization costs of infants.

### 4.3. BPD May Be a Preventable Disease, and the Management of Lung Diseases under Ultrasound Monitoring May Prevent the Occurrence of BPD

In addition to the high incidence of BPD, survivors also had severe near-term (high readmission rate) and long-term (infancy, childhood and adulthood) quality of life problems. This long-term damage includes, but is not limited to, persistent pulmonary structure changes (including pulmonary disorder structure, focal atelectasis, focal bronchiectasis and focal emphysema and diffuse pulmonary fibrosis), persistent respiratory dysfunction (forced vital capacity decrease, the first second forced expiratory volume reduction, 25% to 75% reduction in the first percentile forced vital capacity), and almost 25% of children with airway obstruction, while more than 50% have airway hyperreaction, which can lead to severe breathing problems in infancy, childhood and adulthood. These problems include the presence of significant persistent pulmonary dysfunction, recurrent respiratory tract infections, asthma or asthma-like syndrome symptoms, pulmonary hypertension and exercise intolerance [64,65,66,67,68,69,70,71], which eventually leads to increased readmission rates, and even long-term mortality. In addition to respiratory problems, surviving children with BPD may also have neuropsychiatric problems. Silva et al. [69] conducted a cross-sectional retrospective study of 40 premature infants diagnosed with BPD from 1 January 2014 to 30 December 2015. They were followed up at 6 and 9 months of corrected age, and evaluated with the Denver II Development Scale. They were found to have severe neuropsychomotor development delays, more cognitive impairment and poorer academic progress in childhood or adulthood [70].

We implemented the management of neonatal lung diseases under LUS monitoring and avoided the occurrence of BPD in premature infants by the following measures: (1) Reducing the frequency of ventilator use, shortening the ventilation duration time and avoiding repeated ventilator use. (2) Reasonable adjustment of ventilator parameters. (3) Dynamic monitoring, timely detection of potential MAS, pneumonia, atelectasis and other lung diseases, and timely implementation of bronchoalveolar lavage to remove them [72,73,74]. Therefore, with the popularization and promotion of LUS, the management of newborns under LUS monitoring to avoid the occurrence of BPD has great social benefits, improving the quality of life of the majority of premature infants, and improving the quality of the population. Moreover, it has great economic benefits, and reduces considerable economic expenditure and medical resources for the country. Although the occurrence of BPD cannot be completely avoided, it is possible to significantly reduce its incidence based on LUS care.

### 4.4. Changes in Traditional Pulmonary Disease Management Strategies Greatly Reducing the Mortality of Very Low Birth Weight and Hospitalized Infants

Our results showed that the diagnosis and management of infants under LUS monitoring reduced the mortality and poor prognosis rate of VLBW infants by 85%, and the overall mortality rate of hospitalized infants by 90%. LUS-based care of neonatal lung diseases can reduce the mortality rate of hospitalized infants by adopting the following strategies: (1) Using LUS monitoring to guide mechanical ventilation, ventilator parameter adjustment and weaning from the ventilator to achieve significant results [15]. (2) Optimizing the management of pulmonary hemorrhage and guiding the puncture treatment of hemorrhagic pleural effusion in infants with pulmonary hemorrhage, thus decreasing the fatality rate to 0% [75,76]. (3) Optimizing the management of pneumothorax and guiding the precise puncture treatment of pneumothorax, decreasing the fatality rate to 0% [77,78]. (4) Optimizing the management of RDS, decreasing the case fatality rate to 0% [15,79]. (5) Through the above-mentioned measures, the occurrence of BPD is prevented.

Every year, at least 15~20% of newborn babies need hospitalization; that is, at least 1.8~2 million newborn infants in China receive various kinds of hospitalization treatment every year. Based on the above-mentioned mortality rate of 9.83‰, 18,000 to 20,000 hospitalized newborns die each year in China. After the management of infants under LUS monitoring, the mortality of hospitalized newborns decreased by 90% to less than 1%. As a result, at least 16,000 to 18,000 neonatal deaths could be prevented each year. According to the “Statistical Bulletin on China’s Health Development in 2020” released by the Department of Planning, Development and Information Technology under the National Health Commission, the overall mortality rate of newborns in 2020 is still 3.4‰ [80]. Therefore, it is estimated that more than 40,000 newborns in China face the threat of death every year. The comprehensive application of LUS will certainly play an important role in reducing overall neonatal mortality.

### 4.5. Cost Savings—The Inevitable Side Effects of LUS-Based Care

As described above, LUS-based care has inevitable, remarkable economic effectiveness both for families and states for the following reasons: (1) It reduces the frequency of ventilator use, shortens the duration time of ventilation and reduces the repetition of mechanical ventilation. The cost of ventilator treatment in China is approximately 720 RMB/24 h. Therefore, the decrease in the ventilator use rate and the shortening of the use time will save considerable medical expenses for newborn infants. (2) It avoids or reduces RDS misdiagnosis and reduces the dosage of PS. Exogenous PS is a relatively expensive preparation. (3) It avoids or significantly reduces the occurrence of BPD. In China, the hospitalization cost of each BPD patient is generally more than 200,000 RMB. (4) It shortens the hospitalization time of infants. (5) It significantly reduces infant mortality. All these factors inevitably save considerable medical expenses for both the family and the state.

Regardless, in this paper, according to the results, compared with traditional diagnosis and treatment methods, the use of LUS in the diagnosis of neonatal lung disease and with LUS monitoring of neonatal lung disease management effect is remarkable and improves the diagnostic accuracy and reliability, reducing the misdiagnosis and improving the treatment effects, avoiding the occurrence of BPD, greatly reducing the neonatal mortality, saving medical resources and medical costs, and providing remarkable social benefits. Therefore, it is of great significance to popularize and promote LUS technology worldwide. Particularly in the face of the global economic downturn and the general decline in the birth rate due to the current grim situation, the widespread development of neonatal LUS technology is more urgent. Government health authorities should play an active role in the dissemination of this technology. Of course, the prognosis of an infant does not depend solely on the LUS development status. The overall management levels of the hospitals and the severity of diseases in hospitalized infants also have certain impact on outcomes. However, none of these factors can eliminate the important roles of LUS.

The limitation of this paper is that the data of the LUS-based group are from units with very mature LUS technology, while the data of the control group are all from China. With changes in the environment, relevant indicators will also change accordingly. The degree of proficiency in LUS will influence the effectiveness of the diagnosis and treatment of lung disease. Nevertheless, the results have a reference value for other countries.

## Figures and Tables

**Table 1:** The influence of LUS-based care on the duration neonatal mechanical ventilation ($\bar{x}$ ± sd).

Group	N	TDTV (Days)	T	P
CXR-based care	672	15.03 ± 14.15	17.499	0.000
LUS-based care	357	4.88 ± 3.73		

TDTV:Total duration time of ventilation. LUS: lung ultrasound. CXR: chest X-ray.

**Table 2:** The influence of LUS-based care on RDS fatality rate (%).

Group	N	Death Cases	Fatality Rate (%)	*x* ^2^	P
CXR-based care	282	40	14.2	42.19	0.000
LUS-based care	277	0	0		

RDS: respiratory distress syndrome. LUS: lung ultrasound. CXR: chest X-ray.

**Table 3:** The influence of LUS-based care on fatality rate of pulmonary hemorrhage.

Group	PH	Death Cases	Fatality Rate (%)	*x* ^2^	P
CXR-based care	245	79	32.2	34.86	0.000
LUS-based care	82	0	0		

PH: pulmonary hemorrhage. LUS: lung ultrasound. CXR: chest X-ray.

**Table 4:** The influence of LUS-based care on fatality rate of pneumothorax.

Group	Pneumothorax	Death Cases	Fatality Rate (%)	*x* ^2^	P
CXR-based care	373	41	11.0	7.405	0.007
LUS-based care	61	0	0		

LUS: lung ultrasound. CXR: chest X-ray.

**Table 5:** The influence of LUS-based care on prognosis of VLBW infants.

Group	VLBW	Poor Prognosis Cases	Poor Prognosis Rate (%)	*x* ^2^	P
CXR-based care	2956	1373	46.5	51.595	0.000
LUS-based care	81	5	6.2		

VLBW: very low birth weight. LUS: lung ultrasound. CXR: chest X-ray.

**Table 6:** The influence of LUS-based care on mortality of hospitalized patients.

Group	Hospitalized Patients	Death Cases	Mortality (‰)	*x* ^2^	P
CXR-based care	126565	1244	9.83	40.137	0.000
LUS-based care	5027	5	0.99		

LUS: lung ultrasound. CXR: chest X-ray.

## Data Availability

The datasets used and analyzed are available from the corresponding authors upon reasonable request.

## Supplementary Materials

The following supporting information can be downloaded at: https://www.mdpi.com/article/10.3390/diagnostics12112790/s1, Video S1: Case 1, A infant with respiratory distress. Video S2: Case 2, A infant with respiratory distress.

## Abbreviations

BPD, bronchopulmonary dysplasia; NICU, neonatal intensive care unit; LUS, lung ultrasound; CXR, chest X-ray; RDS, respiratory distress syndrome; TTN, transient tachypnea of the newborn; PS, pulmonary surfactant; VLBW, very low birth weight; MAS, meconium aspiration syndrome; SFS, snowflake-like sign.
